# Neuroimmune Interactions in the Gut and Their Significance for Intestinal Immunity

**DOI:** 10.3390/cells8070670

**Published:** 2019-07-02

**Authors:** David J. Brinkman, Anne S. ten Hove, Margriet J. Vervoordeldonk, Misha D. Luyer, Wouter J. de Jonge

**Affiliations:** 1 Tytgat Institute for Intestinal and Liver Research, Amsterdam UMC, University of Amsterdam, Amsterdam 1105 BK, The Netherlands; 2 Department of Surgery, Catharina Hospital, 5623 EJ Eindhoven, The Netherlands; 3 Galvani Bioelectronics, Stevenage SG1 2NY, UK; 4 Department of General, Visceral-, Thoracic and Vascular Surgery, University Hospital Bonn, 53127 Bonn, Germany

**Keywords:** inflammatory bowel disease, innervation, nerve stimulation, acetylcholine, norepinephrine

## Abstract

Inflammatory bowel diseases (IBD) have a complex, multifactorial pathophysiology with an unmet need for effective treatment. This calls for novel strategies to improve disease outcome and quality of life for patients. Increasing evidence suggests that autonomic nerves and neurotransmitters, as well as neuropeptides, modulate the intestinal immune system, and thereby regulate the intestinal inflammatory processes. Although the autonomic nervous system is classically divided in a sympathetic and parasympathetic branch, both play a pivotal role in the crosstalk with the immune system, with the enteric nervous system acting as a potential interface. Pilot clinical trials that employ vagus nerve stimulation to reduce inflammation are met with promising results. In this paper, we review current knowledge on the innervation of the gut, the potential of cholinergic and adrenergic systems to modulate intestinal immunity, and comment on ongoing developments in clinical trials.

## 1. Introduction

Inflammatory bowel diseases (IBD) are chronic, debilitating conditions that have a major impact on the quality of life for an increasing number of patients worldwide [1]. Extensive experimental and clinical work has been conducted to understand the etiology of IBD and to develop corresponding therapies. The current treatment strategy for IBD is mainly based on a step-up approach, starting with relatively mild immunomodulatory agents such as 5-aminosalicylic acid and steroids, followed by immunosuppressants such as thiopurines and methotrexate, before considering more aggressive drugs (i.e., biologicals), and eventually surgery as a last resort. However, this strategy is not equally effective for all patients and is accompanied by substantial costs and side effects. The necessity to develop novel therapies has ignited innovative views on how to treat IBD and other immune-mediated diseases. In this respect, it is acknowledged that the nervous system is a regulator of immune function which is potentially harnessed to achieve immunosuppression in IBD. Especially, the vagus nerve and its main neurotransmitter acetylcholine (ACh) have been put forward, since vagus nerve stimulation (VNS) was shown to reduce local and systemic inflammation in animal models of endotoxemia, arthritis, and colitis [2,3,4,5]. Therefore, clinical trials are currently being conducted to determine the beneficial effects of VNS in patients [6,7].

The mechanism via which VNS reduces inflammation is yet to be fully elucidated. Although the intestine is densely innervated, it is not clear whether the vagal efferent nerves actually the innervate mucosal cells. This might indicate a role for other types of nerves [8]. Furthermore, it is demonstrated that all sorts of immune cells in the gut could be the target of a wide variety of neurotransmitters, such as ACh, and also epinephrine, norepinephrine (NE), nitric oxide, and a large number of neuropeptides that act as immune modulators. This review aims to delineate the role of neuronal innervation to reduce the progression and relapse of IBD and to indicate potential directions for new developments on neuromodulatory treatments.

## 2. Innervation of the Gut

The autonomic nervous system regulates key functions of the gastrointestinal tract such as motility, secretion, and vasoregulation, and acts autonomously in that its activities are not under direct conscious control. It represents the extrinsic control of the intestine and it is classed as sympathetic and parasympathetic branches based on anatomy and neurotransmitter function. The sympathetic and parasympathetic systems originate in the central nervous system (with cell bodies in the brainstem and spinal cord), while the intrinsic neurons of the enteric nervous system (ENS) reside within the wall of the gastrointestinal tract. The ENS is a distinct part of the central nervous system that acts either independently or in response to external triggers originating from sympathetic and parasympathetic nerves. The ENS is composed of small aggregations of nerve cells, called enteric ganglia, that form nerve fibers innervating effector tissues such as the intestinal muscular layer, blood vessels, and gastroenteropancreatic endocrine cells. It is divided into the myenteric plexus (MP) and the submucosal plexus (SMP). The MP, also known as Auerbach’s plexus, is the outer of the two major ENS plexuses and is comprised of the neurons that are located between the muscle layers of the gastrointestinal tract. It reaches from the esophagus to the internal sphincter and is primarily involved in the regulation of smooth muscle motor patterns such as peristalsis. The SMP (Meissner’s plexus) is the network of neurons located between the muscle layers and the mucosa. Its function is to regulate reflexes like secretion and absorption, as well as the smooth muscle motor function. The SMP is present in the small and large intestines, but it is lacking in the esophagus and the greater part of the stomach. The ENS contains neurotransmitters, such as ACh, and is believed to regulate intestinal immunity [9]. The crosstalk between the gut and the nervous system also comprises neuropeptides that are important mediators between the nervous system and neurons or other cell types in the effector tissues. These small proteins, such as substance P (SP), calcitonin-gene related peptide (CGRP), neuropeptide Y (NPY), vasoactive intestinal polypeptide (VIP), serotonin, somatostatin, and corticotropin-releasing factor, are important in multimodal neuronal communication. Of note, the distribution of these peptides has been widely studied. The abovementioned neuropeptides originate from the dorsal root ganglia [10]. Although a direct link has not yet been established, it is very likely that these molecules act as messengers in the gut-brain axis.

Most of the parasympathetic innervation of the intestine is through the vagus nerve, especially given the recent observation that the sacral preganglionic innervation to the lower gut is sympathetic in nature and not parasympathetic [11]. Preganglionic neurons of vagal efferents originate from the motor neurons of the dorsal motor nucleus and synapse with postganglionic neurons within the MP. The parasympathetic nervous system (PNS) is cholinergic and ACh is the neurotransmitter that is released at both ends which binds to the muscarinic and nicotinic receptors. The distal part of the colon is innervated by pelvic nerves that arise from S2, S3, and S4 nerve roots of the sacral plexus. The stomach and upper gastrointestinal tract are most densely innervated by parasympathetic nerves.

Sympathetic innervation arises in preganglionic fibers at the thoracolumbar intermediolateral nucleus of the spinal cord and synapses with the postganglionic noradrenergic neurons in the prevertebral and paravertebral ganglia at the site of the gastrointestinal tract. The celiac-mesenteric ganglia provide innervation to the stomach, the small intestine, and the proximal part of the large intestine. The remaining part of the large intestine is innervated through the inferior mesenteric ganglia. The rectum is innervated by fibers originating from the pelvic ganglia. In contrast to the vagus nerve, sympathetic nerves spread throughout the entire depth of the gastrointestinal wall where they influence physiological functions such as motility, secretion, and intestinal vasculature [12,13,14] such as the vagal fibers and the sympathetic nerves synapse with the MP and SMP. The main neurotransmitter of the sympathetic nervous system (SNS), NE, binds to the adrenergic GPCR receptors, which contain α and β subunits, and each have several subtypes.

In the early 90s, it was discovered that patients with IBD suffer from autonomic dysfunction [15] which was reflected by a reduced pan-enteric innervation pattern leading to functionally reduced neuronal activity. For instance, inflammation in the intestine is shown to reduce the innervation of the stomach [16]. Innervation of the colon is subject to change following inflammatory processes in the gut mucosa. In line with this, concerning the PNS and SNS, as well as the ENS, colitis is associated with a loss of neurons, an altered neurochemical content, and reduced neurotransmitter release in both animals as patient populations [17]. Moreover, both noradrenergic and cholinergic neuronal pathways mediate stress-induced reactivation of colitis in the rat [18]. This suggests a pivotal role for the autonomic nervous system in regulating intestinal immunity.

## 3. The Anti-Inflammatory Pathway and Cholinergic Modulation of Intestinal Inflammation

### 3.1. The Cholinergic Anti-Inflammatory Pathway

**Figure 1:**
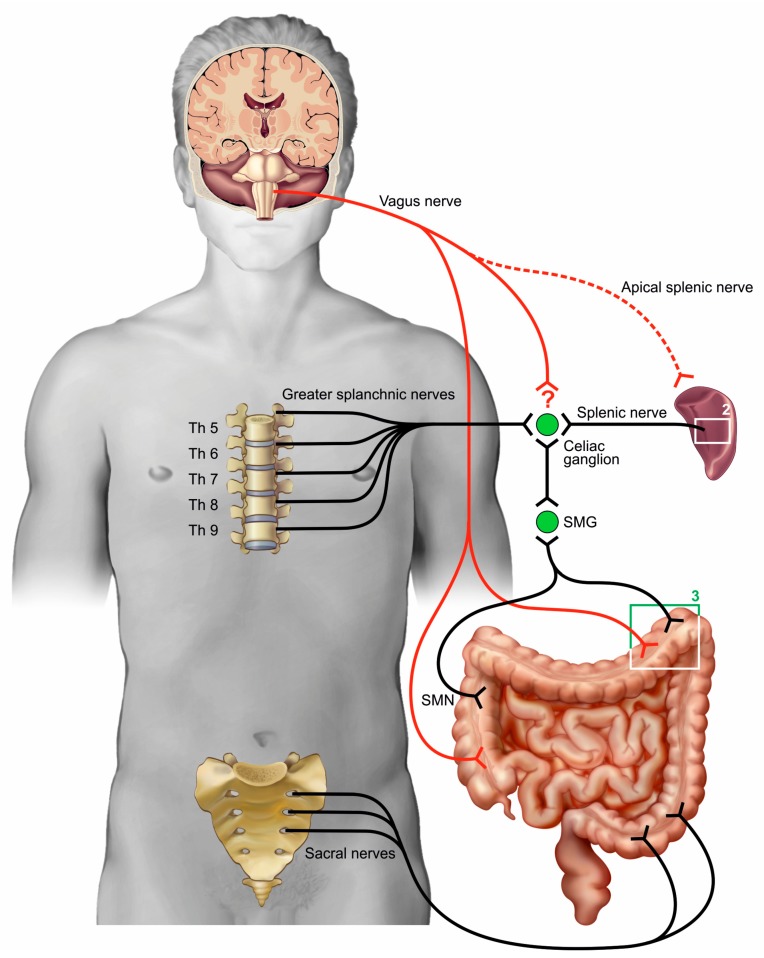
Schematic overview of the existing theories on the (cholinergic) anti-inflammatory pathway. The theory of the cholinergic anti-inflammatory pathway calls for the efferent fibers to suppress inflammation via the splenic nerve bundles. The nerve bundles that innervate the spleen are sympathetic in nature, although cholinergic innervation of the superior pole of the murine spleen was also described (via an apical nerve [20]). Figure 2 displays how the splenic nerve can influence immune cells. It is hypothesized that vagal fibers and the splenic nerve synapse in the celiac ganglion (CG), however, thus far, anatomical studies have not established this. An alternative theory assumes that the greater splanchnic nerves comprise the anti-inflammatory pathway [21]. Both sympathetic and cholinergic nerves innervate the large intestine (Figure 3), although the distal part only receives innervation from the sympathetic nerves that originate from the sacrum. SMG—superior mesenteric ganglion; SMN—superior mesenteric nerves.

The influence of neurons on inflammation in the gut first gained attention when the cholinergic anti-inflammatory pathway (CAIP) was described (Figure 1). It was suggested that this pathway works as a reflex mechanism via which the nervous system controls excessive immune reactions [19]. The mechanism was proposed after it was shown that VNS could attenuate the inflammatory response in an endotoxemia model [5]. Immunity and inflammation are crucial defense mechanisms which protect against potential threats. Obviously, regulation of these processes is essential, since uncontrolled functioning could lead to autoimmune disorders. The nervous system, using rapid nerve signals and promptly acting neurotransmitters, might develop into a control mechanism to sustain excessive inflammatory reactions, leading to a homeostatic immune environment. The etiological reason for such a neuronal immune-regulatory system is supported by the integrative, direct acting, and targeted reactivity that the neuronal system has in favor over humoral immunoregulation, with slow-acting secreted and diffusing mediators. Initially, it was suggested that the CAIP was a clear-cut system where immune cells are targeted by the vagus nerve via its neurotransmitter ACh. However, several anatomical and physiological controversies about the CAIP indicate the need for further elucidation of this theory.

The CAIP is commonly proposed as a reflex mechanism where the vagus nerve functions as both the afferent and efferent part [22]. Various experimental studies have shown that afferent vagal nerve fibers are activated by different stimuli to control inflammation. Afferent fibers react to pathogen-associated molecules, such as endotoxin and viral nucleic acids, as well as to ATP and cytokines which are released by host cells during the course of inflammation [23]. Vagal afferents further contain receptors for hormones such as cholecystokinin, which are released after duodenal intake of high-fat nutrition [24]. The contribution of the efferent part of the vagus nerve is mostly substantiated by the positive effect of VNS in models such as endotoxemia and ileus [5,25]. However, this interpretation overlooks the reality that VNS activates not solely efferent but also afferent fibers. Secondly, it was observed that the spleen was necessary for the anti-inflammatory effect of VNS [26]. This is conflicting, since the spleen is sympathetically innervated and no connections are found between the vagus nerve and the splenic nerve bundles thus far, although superior parts of the spleen receive cholinergic innervation in the mouse [20,27]. The role of the spleen in the CAIP is further discussed below. On the efferent part of the anti-inflammatory pathway, Martelli et al. pointed out that the greater splanchnic nerves are a likely candidate to exert this function rather than the vagus nerve [21,28]. Recently, researchers have shown through electrophysiological experiments that the vagus nerve could indeed activate the sympathetic splanchnic nerves via a route involving the central systems. Strikingly, cutting the splanchnic nerves completely abolished the anti-inflammatory effects of the VNS, suggesting that the splanchnic nerves represent the efferent arm of the anti-inflammatory pathway [29]. Instead of focusing on intervention of the vagus nerve through electrical stimulation, stimulating the bilateral splanchnic nerves might, therefore, be an interesting therapeutic option and should be investigated in future experimental disease models where vagal nerve stimulation has been successful (e.g., RA, IBD, and sepsis models).

### 3.2. VNS in Experimental Colitis and Underlying Mechanisms

The potential anti-inflammatory effects of VNS gave rise to the idea that the vagus nerve is a prospective target for the treatment of IBD. This was substantiated by the fact that vagotomy exacerbated acute and relapsing dextran sulfate sodium (DSS)-induced colitis in rodents [3,30,31]. In these experiments, ventral and dorsal truncal branches of the vagus nerve were cut at the level of the diaphragm. In vagotomized animals, disease outcomes such as histology scores worsened while colonic inflammatory cytokine levels increased. Vagotomy did not change the course of colitis in the macrophage-deficient mice, underlining previous findings that gut macrophages are the end target of the vagus nerve [25,32]. Of particular importance is that vagotomized animals needed a pyloroplasty to sustain food passage, indicating a nonselective effect of the vagotomy [3,30,31]. A more selective approach was recently performed by [33]. In this study, only the vagal branches that project to the intestines were cut. Interestingly, unlike ligation of the vagus branches at the more proximal cervical level, this approach did not affect the severity of colitis [33]. This observation suggests that a vagotomy performed at the more distal level targeting vagal branches supplying the intestine does not suffice to modulate the disease. Probably, vagal immunomodulation of colitis (or IBD) requires other (non-vagal) neural branches that are critically dependent of the cervical vagus, being afferent or efferent in nature.

Hereafter, the potential of the VNS to improve IBD was investigated in several colitis models. In general, a beneficial effect of VNS is shown on colitis outcomes such as disease activity index (DAI), macroscopic and histologic scores and colonic cytokine levels in 2,4,6-trinitrobenzene sulphonic acid (TNBS), and oxazolone-induced colitis [4,34,35,36]. Stimulation parameters vary between experiments, which makes it difficult to compare experiments. The indiscriminate effect of VNS was also shown in these studies, where changes in heart rate following VNS were reported. However, in humans this might be dependent on the location of the stimulator (left or right vagus nerve) [37] and stimulating the vagus nerve below the diaphragm might prevent off-target effects [38]. A detailed overview of the animal studies performed appears in Table 1.

Following the discovery of the anti-inflammatory potential of VNS, mechanistic studies were performed to delineate the underlying mechanism. The vagus nerve mainly exerts its function via the neurotransmitter ACh. Multiple receptors exist for ACh, which are traditionally classified as muscarinic and nicotinic cholinergic receptors, on the basis of their working mechanism. Nicotinic receptors are directly linked to ion channels, whereas muscarinic receptors are G protein-linked receptors that affect cell signaling via, for example, cyclic adenosine monophosphate (cAMP). Following the initial study on VNS, it was demonstrated that VNS was dependent on the α7 nicotinic acetylcholine receptor (α7nAChR) [42]. This receptor is located in the brain but is also present in immune cells such as macrophages, dendritic cells, and T-cells [43,44,45]. When located on neurons, activation causes swift desensitization via the modulation of intracellular calcium. When located on non-neuronal cells, such as immune cells, different mechanisms are described, including classical ion flux, modulation of cAMP or inhibition of p38 mitogen-activated protein (MAP)-kinases, ultimately leading to an inhibition of the release of proinflammatory cytokines such as tumor necrosis factor TNF-α [46]. Since these findings, stimulation of the α7nAChR, both through pharmacological agonists and VNS, has been shown to be potentially beneficial for a wide variety of diseases which include fatty liver disease, kidney ischemia, arthritis, and schizophrenia [6,47,48,49,50]. In colitis models, selective agonists for α7nAChR reduced immune cell infiltration and disease severity, but have also been shown to worsen colitis [51,52,53]. The relevance of α7nAChRs was also demonstrated in a model of postoperative ileus, which is a postsurgical state of intestinal hypomotility with an inflammatory origin [54]. Matteoli et al. showed that surgical inflammation and intestinal transit in mice were improved by VNS. The end targets of VNS were found to be macrophages that express α7nAChR and lie in close proximity to the cholinergic myenteric neurons which are believed to communicate with the vagal efferents [32].

Although the α7nAChR is a plausible target of VNS in sepsis models, vagotomy has also been shown to worsen colitis independent of α7nAChR [31]. Therefore, the influence of other cholinergic receptors in the setting of colitis should not be overlooked. For example, nicotinic receptors, such as α5, are protective in colitis, while VNS improves the phagocytic capacities of intestinal macrophages via α2β4 receptors [55,56]. Colitis could also be ameliorated by muscarinic receptor agonists, although these agonists are believed to primarily act on receptors that are located in the central nervous system [44,57]. In the colonic epithelium, however, colitis affects muscarinic receptors that protect against cytokine-induced barrier dysfunction and maintain intestinal mucosal homeostasis, indicating a local role for muscarinic receptors in immunological homeostasis [58,59,60].

Besides neuronal sources of ACh, immune cells, such as T- and B-cells, potentially participate actively in the cholinergic system and the anti-inflammatory pathway through their production of ACh. This is already extensively reviewed by Fuji et al. [61,62]. In brief, these cells are characterized by the presence of choline acetyltransferase (ChAT), which is the rate-limiting enzyme for the synthesis of ACh. Immunological activation of T-cells upregulates the expression of ChAT, indicating an increase in the production of ACh. This subsequently regulates the immune function, since immune cells express all types of muscarinic receptors (M1–M5) and a vast number of nicotinic receptors. In the intestine, ChAT^+^ T-cells have already been linked to the production of antimicrobial peptides and microbial diversity, although the participation of ACh immune cells in colitis is not clarified yet [63].

## 4. Importance of the Spleen in Mediating the CAIP

In further research on the CAIP, the spleen is recognized as an essential part since splenectomy and ablation of the splenic nerve in rodents abolished the effects of VNS [26,64]. Cutting the splenic nerve bundles also abrogated the positive effects of muscarinic receptor agonists on colitis [57]. Two decades ago, it was demonstrated that splenic nerve activity increases in response to endotoxemia [65]. The sympathetic splenic nerve bundles surround the splenic artery and derive from the greater splanchnic nerves after synapsing in the celiac ganglia. Immunohistochemical studies in rodents revealed that the splenic nerve bundles enter the spleen via the splenic artery and its terminal branches. There, the majority of nerve branches are found in the white pulp, where they form a possible synaptic-like connection with leukocytes [12,66]. Various studies suggest that the spleen is also innervated by parasympathetic fibers, however strong histological evidence for this statement is lacking in the current literature [67]. The participation of the spleen in the anti-inflammatory pathway has given rise to a great deal of debate. Although small branches of the vagus nerve reach the celiac ganglia, no anatomical evidence is found for a connection between the vagus nerve and the splenic sympathetic neurons in tracing studies [27].

**Figure 2:**
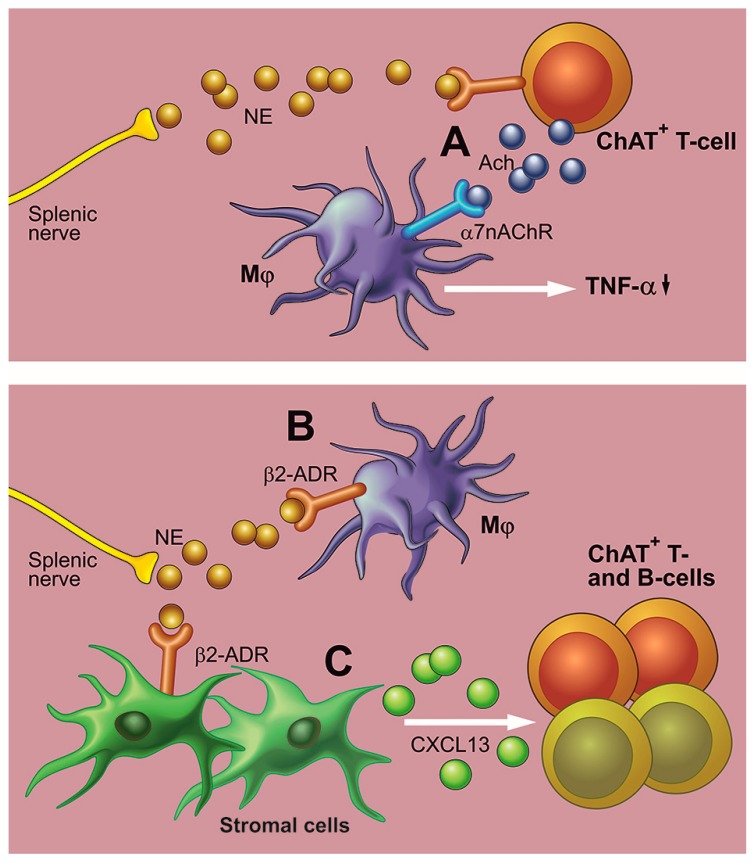
Mechanisms via which stimulation of the splenic nerve controls inflammation. (**A**) Stimulation of the splenic nerve causes the release of norepinephrine (NE), which binds to receptors on the choline acetyltransferase (ChAT)^+^ T-cells. These cells produce acetylcholine (ACh), which reduces the production of inflammatory cytokines such as tumor necrosis factor (TNF)-α by binding to the α7 nicotinic acetylcholine receptor (α7nAChR) of macrophages [68]. (**B**) The released NE directly binds to β2-adrenergic (β2-ADR) on macrophages (or other target cells). (**C**) Upon activation by NE, splenic stromal cells produce chemokines, such as chemokine (C-X-C motif) ligand (CXCL) 13, which control the distribution of ChAT^+^ lymphocytes [72]. Mϕ—macrophage.

ChAT^+^ T-cells provided an explanation for this problem. Rosas-Ballina et al. demonstrated that following VNS, the ChAT^+^ T-cells release ACh in the spleen, thereby possibly relaying the parasympathetic signal [68]. Furthermore, it was found that VNS increased plasma NE via the α7nAChR located on splenic sympathetic neurons. Therefore, it is suggested that ACh released by ChAT^+^ T-cells acts on the α7nAChRs that are located on the splenic sympathetic nerves, which results in the release of NE. This subsequently leads to a reduction of proinflammatory cytokines and systemic inflammation by NE acting on the β-adrenergic receptors on myeloid cells such as macrophages (Figure 2A) [69]. Indeed, immunomodulation via the splenic nerve fibers appears to be dependent on the β-adrenergic receptors and inhibits the production of TNFα independently of α7nAChR [70,71].

Although this is an elegant theory, there should be a clear anatomical connection in the spleen that lies at the basis of the interaction between immune cells and neurons. Rat studies have demonstrated such a relationship, however only a few ChAT^+^ T-cells and B-cells are actually located near the sympathetic neurons in the murine spleen [72]. Possibly, the ChAT^+^ T-cells are affected in an indirect manner via the adrenergic activation of the stromal cells in the spleen expressing CXCL13, a chemokine that recruits B-cells through their receptor C-X-C chemokine receptor (CXCR) 5 [72]. The β-adrenergic activation has previously been shown to control lymphocyte egress in secondary lymphoid organs and might, therefore, be the mechanism via which splenic nerve stimulation could inhibit systemic inflammation [73]. Alternatively, NE that is released by the sympathetic neurons could act directly on the β-adrenergic receptors of the macrophages (Figure 2B,C) [74]. Nevertheless, stimulation of the splenic nerve bundles with either electrical stimulation [20] or ultrasound [75] was able to reduce symptoms in mouse models of rheumatoid arthritis.

Translational studies such as recently performed by Verlinden et al., further clarify whether neurons in the spleen have the potential to influence immune cells [76]. It was already demonstrated that septic patients show a loss of sympathetic splenic nerves, indicating a regulating role in disease [77]. Nevertheless, recent insights also show that the spleen might not be as essential as previously believed, since the anti-inflammatory properties of splanchnic nerve stimulation are spread across other abdominal organs such as the liver and adrenal glands [78].

## 5. Sympathetic Modulation of Gut Immunity

For a long time, it has been recognized that the SNS is a strong modulator of inflammatory activation. Colonic macrophage TNF and IL-6 secretion have been shown to be regulated by sympathetic innervation [79]. Moreover, the anti-inflammatory prospect of sympathetic neurotransmitters, such as epinephrine and NE, has been described in different disease models such as arthritis and sepsis [74,80]. The α- and β-adrenergic receptors are, like cholinergic receptors, present on nearly all types of immune cells. However, there are differences in the expression of these receptors, for example, monocytes show greater β-adrenergic receptor density than lymphocytes [81]. Especially, the β2-adrenergic receptor is involved in the suppression of proinflammatory cytokine release following stimuli such as lipopolysaccharide in vitro and lymphocyte expansion [73,82,83]. The β2-adrenergic receptor stimulation controls inflammation by driving rapid IL-10 secretion [84]. Therefore, NE has gained increasing recognition as a modulator of intestinal inflammation. Moreover, in a DSS-colitis model, sympathetic denervation lead to worsening of the DSS-induced colitis, whereas sympathetic stimulation caused an improvement [85]. This anti-inflammatory effect of NE acting on the β2-adrenergic receptors was highlighted by the finding, in a human study, that patients with IBD who were using β-blockers had an increased risk of developing a disease relapse as compared with IBD patients that were not using β-blockers [57]. Next to this direct anti-inflammatory effect, high concentrations of NE also result in apoptosis in different cell types [86,87,88,89,90]. In lymphocytes, this is mediated, again, by the β2-adrenergic receptor [91]. This also counts as an anti-inflammatory mechanism, in the case that proinflammatory cells are targeted.

Of note, the antagonizing effects of sympathetic activity must be appreciated at the receptor level. In other words, in low concentrations (10^−9^ to 10^−7^ M), NE binds to the α-adrenergic receptors that have proinflammatory properties, whereas in higher concentrations (10^−7^ to 10^−5^ M) NE has more affinity for the β-adrenergic receptors with its anti-inflammatory effects. The opposite applies for adenosine, another neurotransmitter of the SNS, that binds to A1 or A2 adenosine receptors [92]. Together with the facts that sympathetic nerve fibers are lost in inflammatory conditions and NE levels are decreased, a normally present anti-inflammatory β-adrenergic zone becomes a proinflammatory α-adrenergic zone.

The association between the SNS and the inflamed intestine is reciprocal as the intestinal inflammation also affects the nerves and its adrenergic activity. Sympathetic innervation is markedly decreased in inflamed colonic tissue of IBD patients, as well as in various colitis mouse models. In IBD patients, a loss of tyrosine hydroxylase (TH) nerve fiber was demonstrated, and TH was the rate-limiting enzyme for the production of epinephrine and NE. Furthermore, a marked preponderance of proinflammatory SP^+^ fibers was found [40]. The increased presence of nerve repellent factors, such as semaphorin 3C, a protein that exerts repulsive actions against sympathetic fibers specifically, possibly contributes to this differential loss [93]. Fontgalland et al. suggested that somatostatin and NOS, both anti-inflammatory neuropeptides, also interfere with the nerve loss as both somatostatin- and NOS-labeled nerves are reduced in inflamed tissue [94].

The loss of sympathetic nerves in inflamed tissue would logically result in a reduction in sympathetic neurotransmitter levels. Significantly lower NE levels were indeed found in Crohn’s disease patients as compared with healthy controls [95]. This is supported by the fact that the release of NE from sympathetic nerve terminals is restricted in inflamed tissue [96,97,98]. The inflammation induced inhibition could augment to the chronicity of the inflammation as NE negative immune regulation is dampened. Moreover, next to the anti-inflammatory role of the SNS, which is the focus of this review, the main role of sympathetic nerves lies in vasoregulation. Loss of these nerves results in an impaired blood flow, which could add up to sustaining the inflamed environment. The SNS exert opposing proinflammatory and anti-inflammatory functions, depending on the concentration of neurotransmitters and neuropeptides (which is reliant on their release and the presence of sympathetic nerves), the amount and availability of receptors, the receptor affinity, and the timing of sympathetic activity. No consensus exists on the role of the SNS as well as the inflammatory milieu in continuance of the inflammatory processes. Interestingly, a similar discussion on the direction of neuroanatomical change endures in the field of rheumatology [99,100,101]. It has still to be elucidated whether the changes in sympathetic activity are the result of chronic inflammation or vice versa. It could be a combination as the SNS seems to act conflicting. Exerting proinflammatory effects have been observed at the early inflammatory phase and anti-inflammatory in the chronic phase of inflammation [40]. Different experimental models of colitis (acute and chronic) might provide more clarity on the role of the SNS in different stages of the disease. However, both loss of sympathetic activity and local inflammation may lead to an unfavorable situation that supports the ongoing disease processes.

## 6. Neuropeptides

Various subtypes of previously mentioned neuropeptide receptors are also present on immune cells suggesting a possible impact of these molecules on immunity. Due to the anti-inflammatory properties, the therapeutic potential of these have been studied extensively, both in experimental and clinical setting. In TNBS-induced colitis, administration of VIP induced a remarkable amelioration with lower levels of proinflammatory chemokines and cytokines, inhibition of Th1 responses, and induction of a Th2 immune response. Administration of CGRP had a similar effect [102,103]. In rat, CGRP protected the colonic mucosa against TNBS in both the early and late phase of inflammation, while the antagonist of CGRP, i.e., hCGRP, exacerbated TNBS-induced inflammation [104]. Conflicting results on substance P exists with studies showing the beneficial effects in DSS- and TNBS-induced colitis models [105,106] and other studies showing the opposite [107,108]. NPY, one of the most abundant peptides in the autonomic nervous system, and serotonin have a proinflammatory effect [109,110,111,112,113,114]. However, these results have not been supported by clinical evidence, and therefore clinical significance of these results remains unclear.

## 7. Neuronal Innervation and Microbiota

Not only does the SNS impact the intestinal immunity directly, it has also been suggested that there is an association with the gut microbiota. Firmicutes (F) and Bacteroidetes (B) are two major phyla of the domain Bacteria and they dominate the human intestinal microbiota. An increased F/B ratio has been associated with obesity [115] and IBD [116]. Therefore, currently it is being used as marker for pathological conditions [117]. Yang et al. made use of a new bone marrow chimera mouse model that lacked the β-adrenergic receptor 1 or 2 and showed a significant shift in the Bacilli class of Firmicutes in the colon, more specifically in the family of Lactobacillaceae. This was in line with the findings that were published by Bartley et al. [117] that showed reduced β-adrenergic signaling leads to beneficial shifts in the gut microbiota. However, no significant change was found in the F/B ratio. Intriguingly, in both studies the sympathetic depletion lead to a beneficial shift in the gut microbiota composition (i.e., comparable diversity and richness, and decreased Proteobacteria phylum) associated with immune suppression [118]. This is in sharp contrast with the anti-inflammatory effects of NE on cytokine level.

The role of the PNS in modulation of the gut microbiome is still equivocal. Late research in Parkinson’s disease, a common neurogenerative disorder, has demonstrated that atrophy of the vagus nerve represents an important route of disease progression [119]. This pathogenesis has been associated with changes in the gut microbiota composition and inflammation [120,121,122]. However, this could also be the cause of Parkinson disease rather than the result, since VNS did not alter gut microbiota compositions in mice in another study focusing on amyotrophic lateral sclerosis [123] where during surgery the animals of the experimental group received one hour of VNS. Chronic use of VNS might affect the microbiota, and therefore these studies are warranted.

The intestinal microbiome greatly interacts with the mucus barrier. The intestinal epithelium is covered by a dense layer of mucus that functions as the primary defense barrier hosting antimicrobial peptides and preventing bacterial translocation into underlying tissues. Goblet cells (GC) are simple columnar epithelial cells that act as the primary source of mucins that form the mucus layer. Interestingly, ulcerative colitis, not Crohn’s disease, has been associated with a defective colonic mucus layer and a reduced number of GC [124]. Various types of GC exist and their differentiation is based on their location and function. The surface GC secrete mucins continuously to maintain the mucus layer, whereas GC located in the intestinal crypts secrete mucins upon stimulation. The secretion is party controlled by the PNS, since it has been shown that acetylcholine induces a rapid transient increase in mucus secretion in mouse, rat, rabbit, and human colon [125,126,127,128]. The critical role for the autonomic nervous system is stressed by the finding of altered GC differentiation in Hirschprung disease. The congenital aganglionosis leads to an increased GC differentiation and proliferation resulting in changed mucus properties, increasing the susceptibility for inflammation [129]. Taken together, the autonomic nervous system is able to modulate the intestinal microbiota, possibly through the PNS and assured through the SNS.

## 8. The Impact of the Autonomic Nervous System on Intestinal Epithelial Proliferation

Mucosal healing is considered to be a major prognostic factor in the management of IBD. The SNS, as well as the PNS, have been linked to enhanced cell proliferation and tissue regeneration in multiple organs [130]. Recent studies show that the nervous system can also alter intestinal epithelial cell proliferation [131]. However, the mechanism of this effect has not been identified yet. It is either indirectly (via food intake [132] or inflammation as previously described), or directly. The latter could be due to the fact that sympathetic nerves come in close contact with the intestinal epithelium and hence autonomic neurotransmitters bind to the receptors on the proliferating cells in the intestinal epithelium.

Surgical and chemical ablation of autonomic nerves with subsequent loss of neurotransmitters has been associated with an alteration in intestinal epithelial cell proliferation. Various studies have aimed to identify the underlying pathway. However, results are indecisive on whether autonomic neuronal activity would be anti- or pro-proliferative. After SNS or PNS denervation, epithelial cell proliferation has either decreased [130,133,134,135] or increased [136]. Results are time dependent and effects seem to recover due to compensatory mechanisms, such as upregulation of adrenergic or cholinergic receptor expression, compensation by non-denervated branch, and modulation of the ENS [137]. No data exist on a change in epithelial cell proliferation after sympathetic or parasympathetic stimulation, rather than denervation. Potentially, this has a more long-lasting and substantial effect.

**Figure 4:**
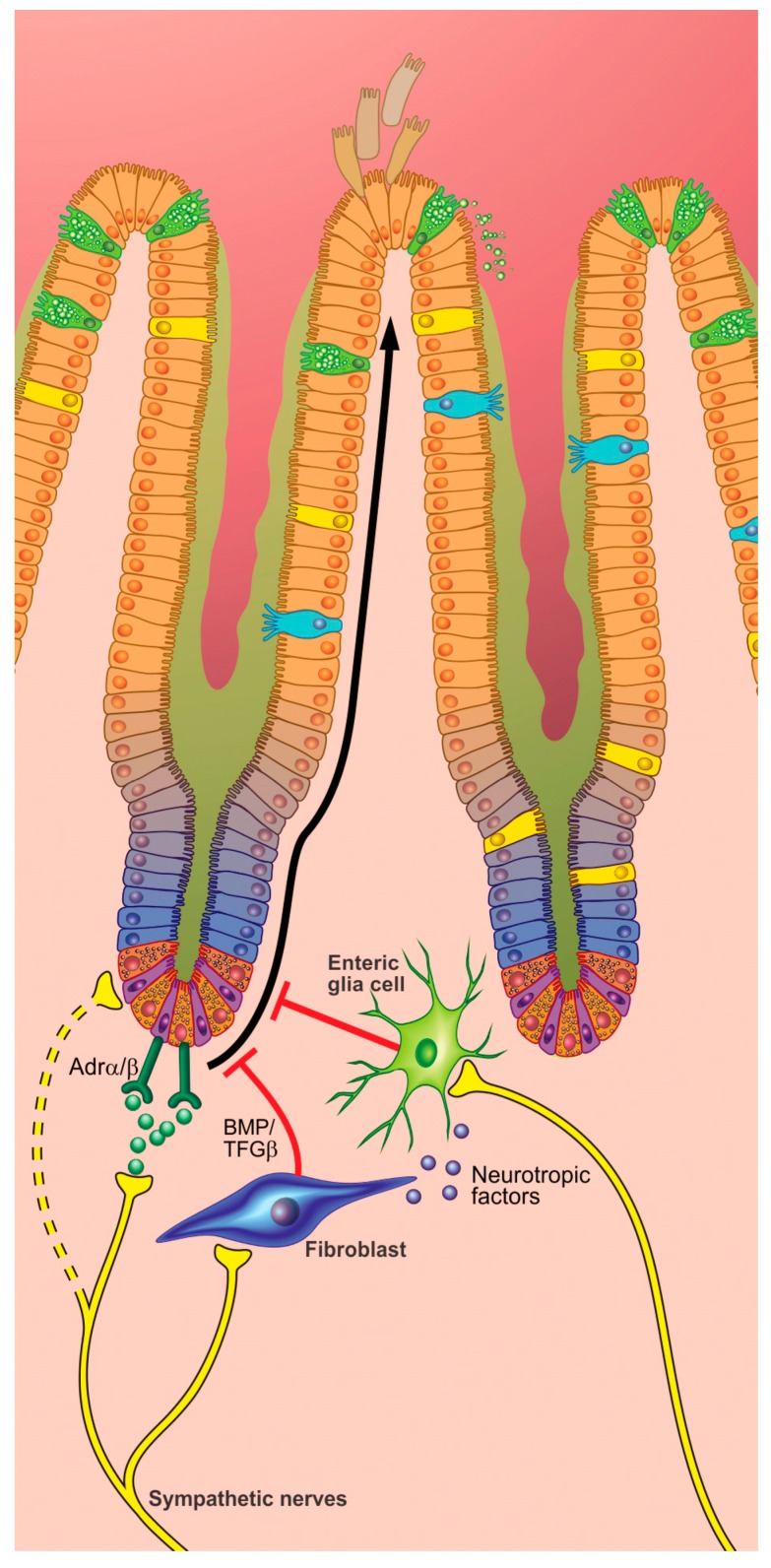
Proposed model of innervation of the intestinal crypt. Sympathetic nerves affect the proliferation in the crypt in multiple ways. Sympathetic neural activity inhibits proliferation through fibroblasts (that produce bone morphogenetic protein (BMP) and transforming growth factor (TGF-β)) and enteric glia cells that express adrenergic receptors. Enteric glia cells also produce neurotrophic factors that are critical in the growth, survival, and differentiation of nerves. In addition, adrenergic receptors are present on cells within the crypt, suggesting that sympathetic neural activity affects the proliferative processes directly.

**Figure 5:**
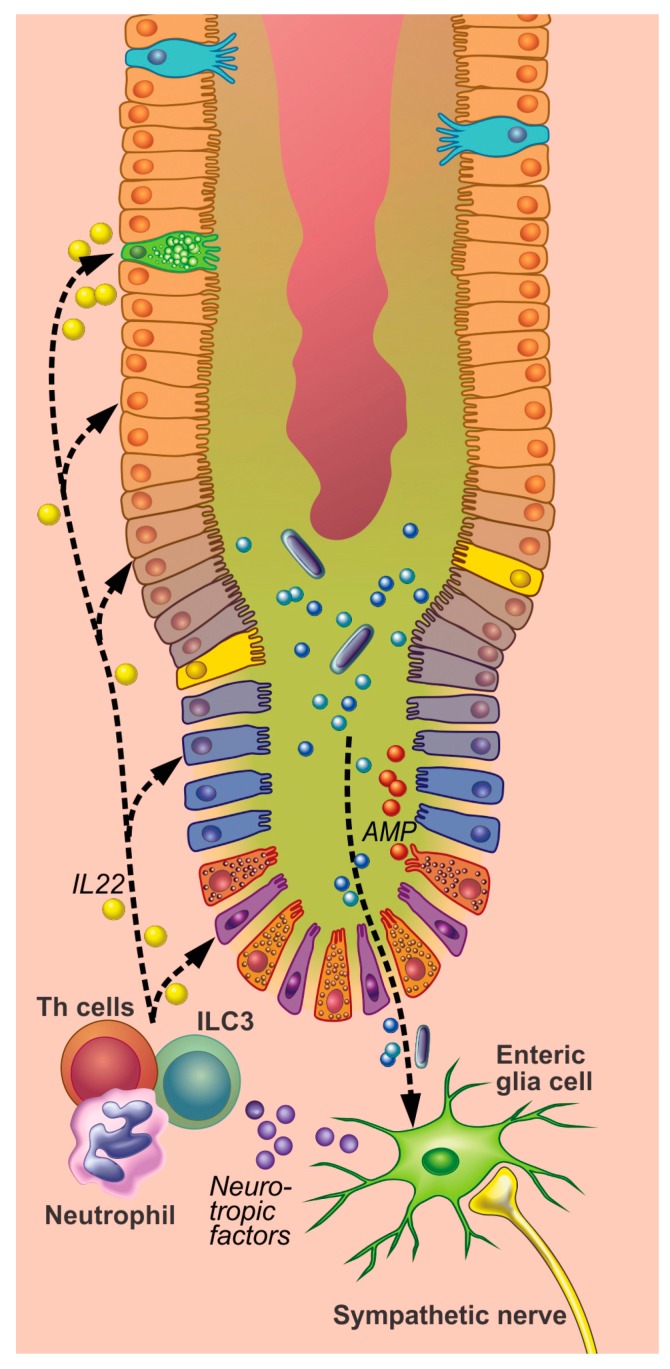
Inflammatory processes in the intestinal crypt. In inflammatory state, enteric glia cells are activated by proinflammatory cytokines and factors like antimicrobial peptides. Under the influence of the sympathetic neural activity neurotrophic factors are produced and extruded. Inflammatory cells, such as neutrophils, T helper (Th) cells, and type 3 innate lymphoid cell (ILC3), interleukin-22 (IL-22) are produced that play a pivotal role in modulating inflammation and stimulating host defense/antimicrobial peptide secretion.

Both sympathetic and parasympathetic neurotransmitter receptors are expressed in the crypt, villus, and epithelial stem cells (Figure 4 and Figure 5) [138,139]. NE and ACh released from these terminals could bind to receptors on stem cells, transit amplifying cells [131], and a combination of these or interact with other cells involved in epithelial cell proliferation pathways. The leucine-rich repeat-containing G-protein coupled receptor (Lgr5)^+^ crypt base columnar cells, which lie deep in the crypts of Lieberkühn are known for their important role in cell proliferation. However, cell types such as Paneth cells, interspersed among the stem cells, or stroma cells, such as myofibroblasts and glia cells, could also take part in these processes, since they are in close proximity. It is likely that they impact epithelial cell proliferation and differentiation partly through the release of cytokines. Cytokines influence the expression of tight junctions and stem cell proliferation, and therefore have a pivotal role in modulating the intestinal epithelial barrier and its underlying cells. This also accounts for other factors, such as neuropeptides. For instance, SP has a pro-proliferative role in epithelial cell growth [140,141]. Its receptors are present in colonic mucosa.

Enteric glial cells (EGC) reside beneath the epithelial layer. Their astrocyte-like shape suggests a communicating role with the central nervous system. Neunlist et al. showed that EGC inhibit intestinal cell proliferation through a transforming growth factor (TGF)-β1-dependent pathway [142]. This antiproliferative effect was underlined by another study that demonstrated an additive upregulation of differentiation-related genes via the activation of the peroxisome proliferator-activated receptor γ, PPARγ [143]. Conversely, in a more recent study, genetic ablation of EGC did not alter intestinal epithelial proliferation. Despite this, it is thought that EGC might influence the epithelium when “activated” by certain infectious or immunological conditions. Glia adopt proinflammatory or anti-inflammatory phenotypes depending on the context [144] and EGC regulate the neurotoxic effects of intestinal inflammation [145]. Given the fact that these cells express β2-adrenergic receptors [146], they could play a role in the proposed interaction between the SNS and epithelial proliferation.

The role of the PNS in epithelial cell proliferation remains debatable, since no direct innervation of the epithelium has been demonstrated yet. It is likely that the ENS plays an important role in this pathway connecting the PNS to the intestinal epithelial barrier through ACh signaling. In addition, denervation of the PNS alters the proliferative rate of the intestinal epithelium while the ENS remains intact [133,134,135,147]. Despite the fact that the ENS, as well as the PNS, exert their functions through ACh, hindering the demonstration of the parasympathetic attribution to epithelial proliferation, which suggests that the PNS can modulate the proliferation directly.

Thus, modulation of parasympathetic activity could have a beneficial effect on proliferative processes in the intestinal epithelium either directly or indirectly via various cell types, and therefore could act as a new therapeutic target in processes such as wound healing in IBD or postoperatively. Further research is needed to enlighten the pathways involved.

## 9. Clinical Studies in the Field of Bioelectronics

The extensive connections between the autonomic nervous system and the intestine together with the prevalence of the intestinal disturbances or diseases that are associated with neuronal activity makes the innervation of the gut an appealing target for new treatment methods. So far, various clinical trials have investigated the use and efficacy of parasympathetic neuromodulatory techniques in the treatment of inflammation. Bioelectronic medicine, mainly represented by VNS, opens new therapeutic avenues for treatment modalities for IBD. It has already proven its efficacy in rheumatoid arthritis [6], sepsis [148], kidney ischemia-reperfusion injury [48], and Crohn’s disease [7,34,35]. Trials on the application of VNS are still ongoing in postoperative ileus, juvenile idiopathic arthritis, and systemic lupus erythematosus. A small clinical trial performed by Bonaz et al. demonstrated that cervical VNS was able to reduce clinical and biological symptoms in five out of seven subjects with active Crohn’s disease [7]. Well-designed, randomized-controlled studies have to be conducted to confirm these promising results. Since cervical VNS also influences physiological functions, such as heart rate, stimulation of the abdominal branches of the vagus nerve might be a more targeted approach [149].

In recent years, developments in noninvasive neuromodulatory techniques have been of interest. Transcutaneous VNS (tVNS) appears promising as no surgical implantation is required. Instead, the auricular concha that is innervated by the vagus nerve is stimulated transcutaneously [150,151]. Several devices have been investigated, such as the Cerbomed NEMOS stimulator (Erlangen, Germany) and the electroCore LLC gammaCore device (Basking Ridge, NJ, USA), which was originally designed to treat primary headache by delivering electrical signals to the cervical part of the vagus nerve. Lerman et al. have shown that tVNS through the use of the gammaCore device decreased cytokine and chemokine levels in healthy individuals [152]. Although the advancement in these noninvasive techniques is encouraging, the risk of noncompliance should be taken into account.

## 10. Concluding Remarks and Future Perspectives

In the last decades, many studies have revealed an important role of the autonomic nervous system in modulating intestinal immunity. Through research on the role of SNS, as well as the PNS, the role of the SNS has been cued as fundamental in the intervention of immune processes. Many open questions however remain. Further research is needed to elucidate the specific cells under the influence of neurotransmitters in both healthy and diseased conditions since the data are conflicting. For instance, specialized epithelial cells, including those present in the crypt stem cell niche, express neurotransmitter receptors, however, whether such cells are truly innervated and functionally affected remains to be established. Local neurotransmitter concentrations critically direct the types of receptors activated, especially in the case of adrenergic receptor classes, further complicating conclusive studies in this field. Nonetheless, it is evident that the autonomic nervous system has great potential to serve as a therapeutic target for inflammatory diseases and to that end the advancement of new neuromodulatory techniques must be pursued. The importance of inflammatory reflexes in regulating acute and chronic intestinal inflammatory disorders is emerging and the ENS appears as a pivotal element linking sympathetic, and more particularly vagal inputs, to the immune system.

Clinically, an added immune regulatory function by neural interfacing may provide us with a powerful tool to enhance remission in IBD patients. Such applications may be more feasible and less invasive than originally thought, making use of implantable devices. Neural interfacing technology provides the basis for mapping neural signals and for bioelectronic medicines. Electrode-based interfaces must be adapted to interrogate visceral nerve activity effectively, but more preclinical work seems necessary to determine this as a true treatment paradigm.

## Figures and Tables

**Figure 3:**
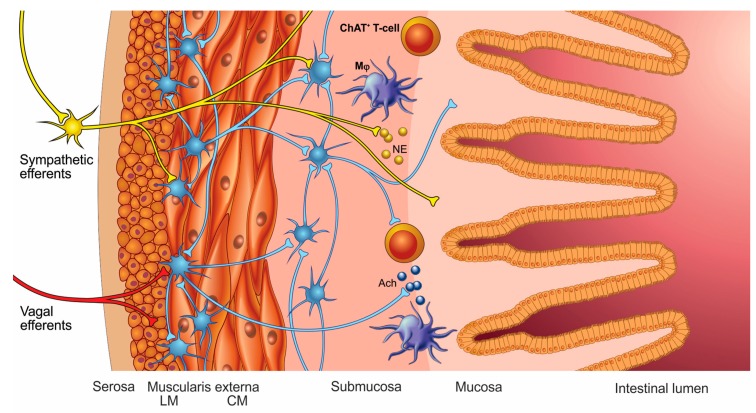
Innervation of the intestinal wall. Both sympathetic and cholinergic nerves innervate the intestinal wall, where they synapse with the enteric nervous system (ENS). Only the sympathetic nerve fibers reach the mucosal layer of the intestine, where they can interact with immune cells. The ChAT^+^ T-cells reside in the intestinal wall, possibly contributing to immunological homeostasis. Mϕ—macrophages; NE—norepinephrine; ACh—acetylcholine.

**Table 1:** Overview of studies that investigated cutting the nerves or nerve stimulation in models of colitis.

Type of Stimulation or Denervation	Colitis Model	Species	Study	Location	Stimulation Details	Main Outcomes
Vagotomy	Acute DSS	C57BL/6 mice	Ghia 2006 [3]	Sub-diaphragmatic	-	DAI ↑
						Macroscopic score ↑
						Histology score ↑
						MPO ↑
						Colonic cytokines ↑
			Di Gio-vangiulio 2016 [31]	Sub-diaphragmatic	-	DAI ↑
						Weight loss ↑
						Survival rate ↓
			Willemze 2018 [33]	Intestine specific (celiac branch vagus)	-	DAI =
						Weight loss =
						Colonic cytokines =
	Relapsing DSS	C57BL/6 mice	Ghia 2007 [30]	Sub-diaphragmatic	-	DAI ↑
						Macroscopic score =
						Histology score ↑
						MPO ↑
						Colonic cytokines ↑
VNS	TNBS	Sprague-Dawley rats	Meregnani 2011 [34]	Left cervical vagus	3h per day 1 mA, 5 Hz, 500 μs, 10 s ON, 90 s OFF	Weight loss ↓
						Histology score ↓
						Colitis index ↓
						MPO ↓
						Colonic cytokines =
			Sun 2013 [35]	Left cervical vagus	3h per day 0.25 mA, 20 Hz, 500 μs, 30 s ON, 5 min OFF	DAI ↓
						Weight loss ↓
						Macroscopic score ↓
						Histology score ↓
						MPO ↓
						Colonic cytokines ↓
			Jin 2017 [36]	Left cervical vagus	3h per day 1–3 mA, 5 Hz, 500 μs 10 s ON, 90 s OFF	DAI ↓
						Weight loss ↓
						Macroscopic score ↓
						Histology score ↓
						MPO ↓
						Plasma cytokines ↓
			Payne 2019 [38]	Sub-diaphragmatic	3h per day 1.6 mA, 10 Hz, 200 μs 30 s ON, 5 min OFF	Stool score↓
						Blood in stool ↓
						Plasma C-reactive protein↓
						Histology score↓
						Intestinal leukocyte infiltration↓
	Oxazolone	Balb/c mice	Meroni 2018 [4]	Right cervical vagus	5 min per day 1 mA, 5 Hz, 1000 μs	Survival rate ↑
						Histology scores =
						Colonic and serum cytokines ↓
Sympathec-tomy	TNBS	Sprague-Dawley rats	McCaf-ferty 1997 [39]	Systemic (6-OHDA)	-	Macroscopic score ↓
						Histology score ↓
						MPO ↑
	Acute DSS	BALB/c mice	Straub 2008 [40]	Systemic (6-OHDA)	-	Colon length ↑
						Histology score ↓
		C57BL/6 mice	Willemze 2018 [33]	Superior mesenteric nerve	-	DAI ↑
						Weight loss =
						Colonic cytokines =
	Relapsing DSS	BALB/c mice	Straub 2008 [40]	Systemic (6-OHDA)	-	Colon length ↓
						Histology score ↑
	Spontaneous	IL10 -/- mice	Straub 2008 [40]	Systemic (6-OHDA)	-	Histology score ↑ Colonic cytokines ↑
		RAG1 -/- mice	Willemze 2019 [41]	Systemic (6-OHDA)	-	Weight loss =
						Colon weight =
						Histology score ↑
						Colonic cytokines =
				Superior mesenteric nerve	-	Weight loss =
						Colon weight ↑
						Histology score ↑
						Endoscopy score =
						Colonic cytokines ↑
SNS	Acute DSS	Sprague-Dawley rats	Willemze 2018 [33]	Superior mesenteric nerve	5 min twice daily 0.2 mA, 10 Hz, 2000 μs	DAI ↓
						Weight loss =
						Histology =
						Colonic cytokines =
						Endoscopy score =

VNS—vagus nerve stimulation; SNS—sympathetic nerve stimulation; DSS—dextran sulfate sodium; TNBS—trinitrobenzenesulfonic acid; DAI—disease activity index; MPO—myeloperoxidase; 6-OHDA—6-hydroxydopamine.
